# Birthweight and pregnancy outcomes in obese class II women with low weight gain: A retrospective study

**DOI:** 10.1371/journal.pone.0215833

**Published:** 2019-05-20

**Authors:** Estelle Roussel, Salma Touleimat, Laurence Ollivier, Eric Verspyck

**Affiliations:** 1 Department of Obstetrics and Gynecology, Belvedere Maternity Hospital, Mont-Saint-Aignan, France; 2 Department of Obstetrics and Gynecology, Rouen University Hospital, Rouen, France; 3 Normandie University, UNIROUEN, EA “NeoVasc”, Laboratory of Microvascular Endothelium and Neonate Brain Lesions, Rouen, France; Bradford Institute for Health Research, UNITED KINGDOM

## Abstract

**Introduction:**

To determine if weight gain below the institute of medicine (IOM) guidelines improves pregnancy outcomes and influences birthweight for women with class II obesity.

**Materials and methods:**

We retrospectively included 996 women with class II obesity with singleton gestations and delivered at term in two hospitals providing level III maternal care between January 2006 and December 2015. Women were classified into three groups: weight gain within IOM recommendations (≥5-≤9kg), low weight gain (≥0-<5kg), and weight loss (<0kg). Maternal complications and birth weight were reported in all groups. The group presenting weight gain within IOM recommendations was considered as the reference group.

**Results:**

424 women (42.5%) constituted the reference group and presented weight gain within IOM recommendations; whereas 370 (37.1%) presented low weight gain and 202 (20.3%) presented weight loss. The rate of birthweight above 4000 g was reduced in women with low weight gain (odds ratio (OR) = 0.62 [0.42–0.93]; *p* = 0.02) and in women with weight loss (OR = 0.58 [0.35–0.96]; *p* = 0.02). However, the rates of small for gestational age fetuses (SGA) < 10^th^ percentile was increased in women with weight loss (OR = 1.63 [1.03–2.58]; p = 0.02). Maternal and neonatal complications were not significantly different between groups.

**Conclusion:**

While low weight gain in women with class II obesity may reduce macrosomia without excessive risk of SGA, it has no effect on maternal and neonatal complication rates.

## Introduction

The prevalence of maternal obesity is increasing in developed countries and estimated to be around 12% in France [1]. Maternal complications associated with obesity are increased rates of cesarean section, preeclampsia, gestational diabetes and post-partum haemorrhage (2). Neonatal complications associated with maternal obesity are mainly linked to macrosomia, which in turn increases the prevalence of shoulder dystocia, neonatal asphyxia and traumatic lesions related to delivery [2]. In addition, fetal complications i.e. prematurity, stillbirth, congenital abnormalities, and childhood and adolescent metabolic disorders are also more frequently reported in obese women [2].

The prevalence of class I obesity, represented by a body mass index (BMI) between 30 and 34.9 kg/m^2^, as well class II obesity (BMI 35–39.9) and class III obesity (BMI≥40) has increased over the past few decades in countries with high medical resources [1,3]. However, the prevalence of women with class II and III obesity remain low in France with respectively 3.7% and 1% of the overall population [1]. In addition, various maternal and neonatal complications are more frequently reported in cases of class II and III obesity in comparison with class I obesity [4–6].

The Institute of Medicine (IOM) recommends a lower weight gain (less than 9 kg) in obese pregnant women (BMI ≥ 30 kg/m^2^) than in women within normal weight range (18.5–24.9 kg/m^2^) [7]. Evidence suggests there is an increased risk of maternal complications and fetal macrosomia in pregnant obese women with weight gain above IOM guidelines i.e. more than 9 kg [8–10]. However, the IOM did not specify if an optimal lower weight gain should be adapted to the class of obesity; i.e. if women with class II and III obesity should have lower weight gain than women with class I obesity. In that way, several authors have shown that a weight gain inversely proportional to the severity of obesity could be associated with a decrease of macrosomia and maternal complication rates [8–10]. The purpose of this paper was to evaluate the maternal and neonatal complications in a population of obese women without excessive weight gain (above 9 kg) as recommended by IOM and ACOG guidelines [2,7]. We also focused this study in obese women with a medium increased health risk i.e. class II obesity due to the expected difficulties in recruiting women with class III obesity. The objective of our study was to retrospectively assess if weight gain below IOM recommendations could reduce maternal and neonatal complications in women with class II obesity.

## Materials and methods

This retrospective study included obese women followed up in two hospitals providing level III (Rouen University Hospital) and II (Belvedere General Hospital) maternal care belonging to the same territorial health care system and using similar protocols of care. Data were recorded retrospectively using computer-based charts for 10 years starting on the 1^st^ of January 2006 and ending on the 31^st^ of December 2015. Data were collected prospectively and systematically during the pregnancy follow-up by doctors or midwives. Inclusion criteria were: singleton pregnancy, term delivery (between 37 and 41 weeks of gestation), and a BMI comprised between 35 and 40 kg/m^2^. Women were excluded if their weight gain exceeded 9 kg, if the last weight measure was recorded more than a week preceding delivery and in cases of incomplete/missing data. The Committee for the Protection of Person Nord-Ouest I has examined this work and found it conforms to the ethical standards and to the scientific requirements applicable to biomedical research. Date of approval: 26 March 2018. Reference number: E2018-26

Maternal weight was systematically recorded during regular pregnancy follow-up and pregnancy weight gain was calculated by deducting self-reported pre-pregnancy weight from the weight recorded at the last prenatal appointment. BMI was calculated using self-reported prepregnancy weight in kilograms and height in centimetres. Small-for-gestational-age (SGA) new-borns were defined as those whose birth weight was under the 10^th^ percentile, adjusted using the French standard references to the sex of the newborn and to maternal characteristics i.e. parity and pre-pregnancy height and weight [11]. The same standards were used to define large-for-gestational-age (LGA) newborns, characterised by a birthweight above the 90^th^ percentile. Macrosomia and small birth weight were defined by a birth weight above 4000g and below 2500g, respectively.

Maternal characteristics recorded systematically in the charts included pre-gestational BMI, maternal age, parity, ethnic group, marital status, occupation as well as cigarette smoking during pregnancy. Concerning medical history, the relevant elements recorded were pre-existing diabetes and hypertension. During pregnancy, the occurrence of gestational diabetes, gestational hypertension, preeclampsia, postpartum hemorrhage (blood loss more than 500 ml) or severe postpartum hemorrhage (blood loss more than 1000 ml) as well as the term and mode of delivery were reported. Gestational diabetes was systematically identified using a 50g oral glucose challenge test and subsequent 100g oral glucose tolerance test in women with a positive (140 mg/dl) 50g screen or only 75 mg oral glucose tolerance test [12]. When gestational diabetes was discovered, it was managed according to French guidelines [12]. Briefly, women received a specific treatment of gestational diabetes i.e. dietetics, physical exercise, blood glucose self-monitoring, and insulin therapy if appropriate by a diabetologist. In the other hand, gestational hypertension and preeclampsia were managed following international recommendations [13]. Ultrasound examination was recommended for each trimester of pregnancy and the third trimester ultrasound was performed between 30 and 35 weeks of gestation. Additional ultrasound examinations were performed when the estimated fetal weight was above the 90^th^ percentile or below the 10^th^ percentile, regardless of the presence of obesity or of the amount of maternal weight gain. Indications for labour induction were in accordance to the French Healthcare Authority guidelines [14]. Main neonatal characteristics recorded were sex, birth weight, Apgar score at 5 minutes, umbilical arterial pH at birth, admission to neonatal unit, and death.

### Statistical analysis

Women included in the study were classified into three groups according to their weight gain during pregnancy: IOM recommended weight gain (≥ 5-≤ 9 kg), low weight gain (≥0-<5 kg) and weight loss (<0 kg). The number of subjects necessary to be included in the study was calculated *a priori*, given that the expected percentage of SGA fetuses is around 7% in women presenting a weight gain within IOM recommendations, while it is around 15% in women presenting weight loss (8). In order to show a statistically significant difference between groups with a statistical power of 80%, 240 women had to be included in each group. Maternal and neonatal characteristics were compared between groups using a χ^2^ test for qualitative variables, and Kruskal Wallis test for qualitative variables. A logistic regression was performed to calculate odd ratios (OR) of numerous events in women presenting low weight gain or weight loss, considering women with recommended weight gain as the reference population. The maternal and neonatal events for which OR were calculated were gestational diabetes, gravidic hypertension, preeclampsia, spontaneous labour, cesarean section (CS), postpartum hemorrhage, SGA < 3^rd^ and < 10^th^ percentile, LGA > 90^th^ and > 97^th^ percentile, birth weight < 2500 g, macrosomia with birth weight > 4000 g, severe macrosomia with birthweight > 4500 g, Apgar score at 5 minutes, umbilical arterial pH at birth, and admission to neonatal unit. Maternal age, parity, ethnic group, chronic hypertension, pre-existing diabetes mellitus, and tobacco use were the confounding factors taken into account in the logistic regression. A difference between groups was considered statistically significant when the p value was strictly inferior to 0.05, with a confidence interval of 95%. Statistical analysis was conducted using Stata 8.0 software.

## Results

**Fig 1:**
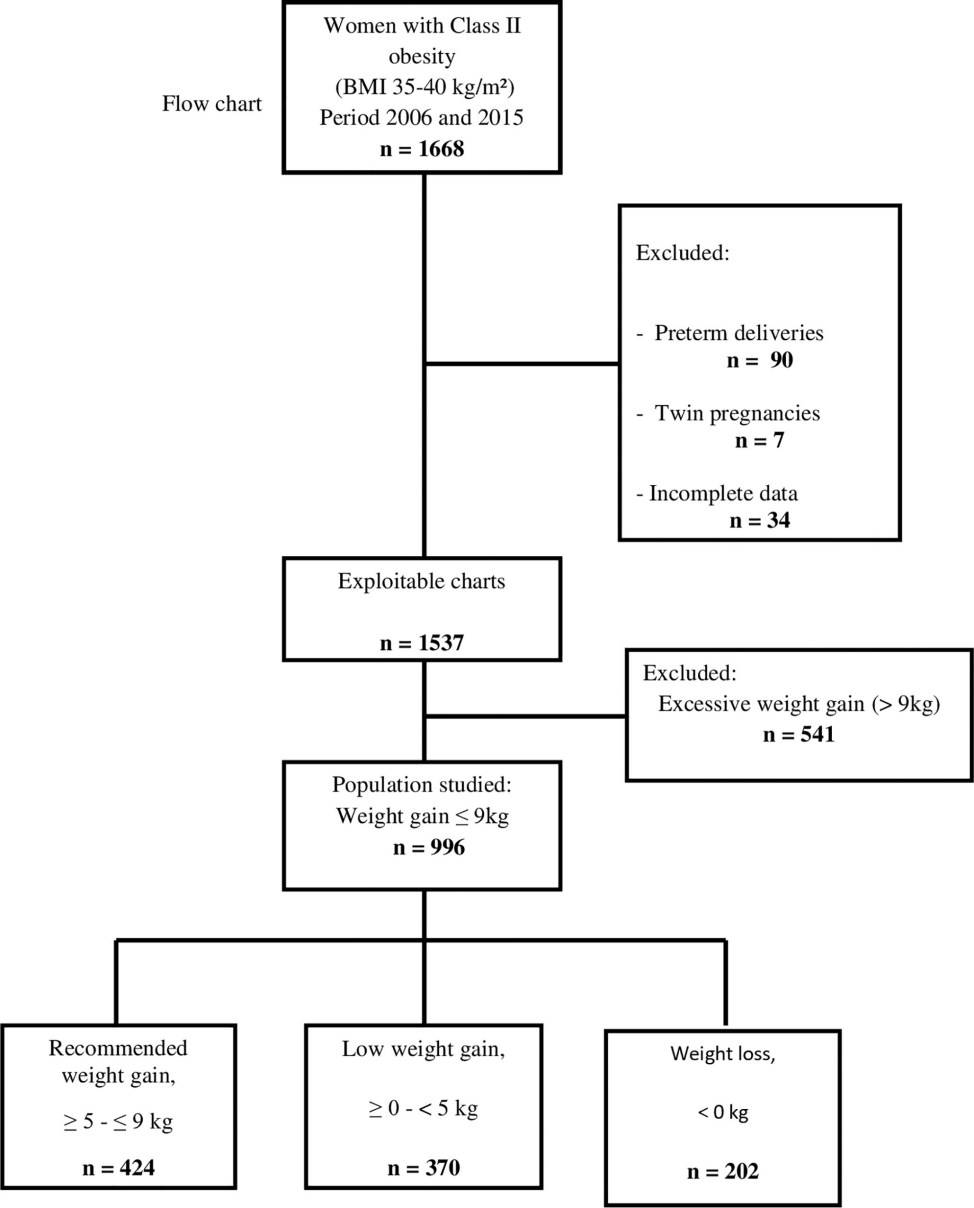
Flow chart.

During the study period, 1668 pregnant women with class II obesity were followed up and 996 of them were included in the study (Fig 1). Exclusion criteria were pregnancy weight gain of more than 9kg (n = 541), preterm delivery (n = 90), multiple pregnancies (n = 7), and incomplete medical charts (n = 34). Among these 996 women, 424 (48.6%) presented a weight gain within IOM recommendations, 370 (37.1%) presented low weight gain, and 202 (20.3%) presented weight loss.

Table 1 presents maternal and neonatal characteristics, as well as obstetrical complications with respect to maternal weight gain. Maternal characteristics were comparable between groups except for BMI and activity. Among the 996 women included in the study, 257 (25.8%) had a cesarean section and 22 (2.2%) presented with preeclampsia. The rate for Gestational diabetes was 18.3% (n = 183) in the overall sample and 40.9% (n = 75) of them were treated with insulin therapy. Concerning birth weight, there was a higher incidence of macrosomia in women with recommended weight gain (18.6%), while SGA occurred more often in women presenting weight loss (19.8%). Neonatal outcomes were comparable between the three groups. No maternal deaths were reported during the study period. One infant death was reported.

**Table 1:** Maternal and pregnancy characteristics of women with class II obesity by gestational weight gain.

	Recommended weight gain, ≥ 5 - ≤ 9 kg (n = 424) [48.6%]	Low weight gain, ≥ 0 - < 5 kg (n = 370) [37.1%]	Weight loss, < 0 kg (n = 202) [20.3%]	p
**Maternal characteristics**				
Maternal age (years)	29.24 ± 5.1	28.94 ± 5.5	29.48 ± 5.2	0.48
Body Mass Index (Kg/m2)	36.88 ± 1.4	37.17 ± 1.4	37.44 ± 1.3	0.01
French origin	335 (79)	294 (79.4)	154 (76.2)	0.64
Married	(41.7)	146 (39.4)	90 (44.6)	0.49
Occupation	247 (58.3)	209 (56.5)	93 (46)	0.01
Smoking	80 (18.9)	75 (20.3)	46 (22.8)	0.52
Rouen University hospital	210 (48.5)	173 (46.6)	91 (45)	0.53
**Obstetrical and medical history**				
Nulliparous	111 (26.2)	119 (32.2)	48 (23.8)	0.06
Pre-existing diabetes	10 (2.4)	5 (1.4)	3 (1.5)	0.55
Chronic hypertension	24 (5.7)	11 (3.0)	5 (2.5)	0.08
Previous cesarean section	88 (20.8)	72 (19.5)	36 (17.8)	0.68
**Pregnancy Outcomes**				
Gestational Diabetes	73 (17.2)	69 (18.6)	41 (20.3)	0.71
Preeclampsia	13 (3.5)	6 (1.6)	3 (1.5)	0.15
Cesarean delivery	121 (28.5)	91 (24.6)	45 (22.2)	0.19
Severe perineal tears	1 (0.33)	1 (0.35)	1 (0.63)	0.85
Postpartum hemorrhage	30 (7.1)	35 (9.4)	12 (5.9)	0.26
Gestational age (weeks)	39.6 ± 1.1	39.6 ± 1.2	39.6 ± 1.1	0.81
Birthweight (grams)	3554 ± 497.5	3450 ± 469.9	3431 ± 490.8	0.001
Male sex	219 (51.6)	188 (50.8)	94 (46.5)	0.47
Small for Gestational Age	55 (13.0)	48 (13.0)	40 (19.8)	0.04
Large for Gestational Age	51 (12)	31 (8.4)	17 (8.4)	0.16
Macrosomia (> 4000g)	79 (18.6)	46 (12.4)	24 (11.9)	0.02
Low birth weight (< 2500g)	6 (1.4)	7 (1.9)	6 (3.0)	0.41
Apgar score at 5 min < 7	4 (0.9)	7 (1.9)	4 (1.9)	0.45
Umbilical arterial pH < 7.1	16 (5.0)	8 (2.7)	2 (1.2)	0.10
Admission to neonatal unit	6 (1.4)	6 (1.1)	6 (2.9)	0.37
Neonatal death	0	0	1	

Date are mean ± standard deviation, n (%)

Small-for-gestational-age = new-borns with birth weight under the 10^th^ percentile, large-for-gestational-age = new-borns with birthweight above the 90^th^ percentile

Maternal complications analysed using logistic regressions are presented in Table 2. The incidence of gestational diabetes, gestational hypertension, preeclampsia, postpartum hemorrhage, or cesarean section in women with low weight gain or weight loss compared to women with recommended weight gain were not significant.

**Table 2:** Maternal outcomes associated with gestational weight gain or loss in women with class II obesity†.

Maternal outcomes	Recommended weight gain ≥ 5 - ≤ 9 kg (n = 424 [48.6%])	Low weight gain ≥ 0 - < 5 kg (n = 370 [37.1%])	Weight loss < 0 kg (n = 202 [20.3%])
Gestational diabetes	1.0	1.26 (0.76–1.89)	1.03 (0.59–1.81)
Gravidic hypertension	1.0	0.95 (0.36–2.50)	0.40 (0.08–1.90)
Preeclampsia	1.0	0.47 (0.17–1.26)	0.49 (0.13–1.78)
Spontaneous labour	1.0	0.94 (0.54–1.66)	1.47 (0.83–2.69)
Cesarean section	1.0	0.85 (0.61–1.17)	0.76 (0.51–1.44)
Scheduled cesarean section	1.0	1 .11 (0.73–1.70)	0.89 (0.52–1.52)
Repeated cesarean section	1.0	1.28 (0.77–2.13)	1.00 (0.53–1.87)
Postpartum hemorrhage	1.0	1.36 (0.81–2.28)	0.84 (0.42–1.71)
Severe postpartum hemorrhage	1.0	1.64 (0.77–3.53)	0.52 (0.14–1.88)

Data are adjusted odds ratio and 95% confidence intervals for low weight gain and weight loss current recommended weight gain

†Adjusted for maternal age, parity, ethnicity, chronic hypertension, pre-existing diabetes mellitus, and tobacco use

Neonatal weight characteristics analysed by logistic regression are presented in Table 3. The incidence of macrosomia was significantly reduced in both women presenting low weight gain and weight loss, compared to those presenting recommended weight gain (OR = 0.62 (0.42–0.93), p = 0.02, and OR = 0.58 (0.35–0.96), p = 0.02, respectively). On the other hand, the incidence of SGA newborns (birthweight <10^th^ percentile) was significantly increased in women presenting weight loss (OR 1.62 (1.02–2.58), p = 0.02) compared to those with recommended weight gain, while it was not modified in women presenting low weight gain (OR = 0.96 (0.63–1.47)). No particular neonatal risks were reported related to maternal weight gain.

**Table 3:** Birthweight and neonatal outcomes associated with gestational weight gain or loss in women with class II obesity†.

Birthweight outcomes	Recommended weight gain, ≥ 5 - ≤ 9 kg (n = 424 [48.6%])	Low weight gain, ≥ 0 - < 5 kg (n = 370 [37.1%])	Weight loss, < 0 kg (n = 202 [20.3%])
Low birthweight < 2500g	1.0	1.27 (0.40–3.99)	2.32 (0.70–7.71)
SGA <10^th^ percentile	1.0	0.96 (0.63–1.47)	**1.62 (1.02–2.58)**
SGA < 3^th^ percentile	1.0	1.09 (0.50–2.33)	1.74 (0.78–3.87)
LGA > 90^th^ percentile	1.0	0.67 (0.42–1.08)	0.64 (0.36–1.16)
LGA > 97^th^ percentile	1.0	0.46 (0.20–1.02)	0.58 (0.23–1.47)
Macrosomia > 4000g	1.0	**0.62 (0.42–0.93)**	**0.58 (0.35–0.96)**
Macrosomia > 4500g	1.0	0.43 (0.16–1.13)	0.74 (0.28–1.96)
Apgar score at 5 min < 7	1.0	1.78 (0.51–6.22)	1.91 (0.46–7.09)
Umbilical arterial pH < 7.1	1.0	0.54 (0.22–1.32)	0.27 (0.06–1.22)
Admission to neonatal unit	1.0	1.08 (0.33–3.47)	2.35 (0.72–7.60)

Data are adjusted odds ratio and 95% confidence intervals for low weight gain and weight loss current recommended weight gain

†Adjusted for maternal age, parity, ethnicity, chronic hypertension, pre-existing diabetes mellitus, and tobacco use

Abbreviations: SGA, small for gestational age; LGA, large for gestational age

## Discussion

This study demonstrates that gaining less than IOM guidelines may reduce the risk of macrosomia without increasing the risk of small for gestational age in pregnant women with class II obesity. However, no beneficial effect of a low pregnancy weight gain was observed on maternal and neonatal outcomes in obese class II women at term.

The main strength of this study is that we report here for the first time the findings from a large cohort of women with class II obesity with various maternal and neonatal outcomes. The number of women to include was also calculated *a priori* according to the statistical power necessary to study various birth weight outcomes depending on maternal weight gain during pregnancy. In this study, we report neonatal birth weight percentiles after adjustment for maternal and neonatal parameters extracted from previous French epidemiologic data.

Nevertheless, our study has several weaknesses. Firstly, the retrospective design of the study could lead to residual confounders and misclassification bias. Secondly, we did not analyzed women with class III obesity due to their very low reported incidence in our maternities. Finally, the study was underpowered to detect some rare adverse neonatal outcomes i.e. neonatal death or birth asphyxia.

The IOM guidelines issued in 2009 recommend a pregnancy weight gain of 5 to 9 kg for all obese women and regardless of their class of obesity [7]. The main justification for this recommendation was that both low pregnancy weight gain and pregnancy weight loss could be associated with an increased incidence of intra-uterine growth retardation (IUGR) [15,16]. However, the IOM recognises that lower weight gain in women with class II and III obesity could have some beneficial effects on women and newborns [17]. To date, there are few data available on pregnancy weight gain below IOM recommendations and whether this could improve maternal and neonatal outcomes in women with class II and III obesity. A large retrospective American study showed a significant decrease in the incidence of preeclampsia, cesarean section deliveries and LGA newborns in women with class II and III obesity when maternal weight gain was lower than IOM recommendations at term [8]. The authors of this study demonstrated that pregnancy complications were significantly reduced when maternal weight gain/loss in women with class II and III obesity was between <5 kg to 0 and 0 to <-5 kg, respectively. Moreover, the risk of SGA newborns was only increased when maternal weight loss exceeded ≥ - 5kg. However, this study had several limitations resulting from incomplete data on pregnancy outcomes and issued solely from birth certificates. In addition, SGA and LGA newborns were only defined according to term of birth and ethnic origin but without taking into account neonatal sex, maternal height or pre-pregnancy BMI. It is well known today that using birth weight standards without considering neonatal sex could overestimate the incidence of SGA in female neonates [15]. Moreover, including maternal characteristics in birth weight standards could better select SGA newborns at risk of neonatal complications [11]. Finally, our study show**s** that the incidence of SGA newborns increased only in women who lost weight during their pregnancy and this finding was coherent with previous literature [10]. However, the incidences of severe SGA < 3^rd^ percentile and adverse neonatal outcomes were not increased in this population of women.

In contrast with *Kiel et al*., the findings of our study do not show a decrease in pregnancy complications in women with low weight gain or weight loss. This may be due to the low statistical power of our study mainly related to our smaller cohort. In addition, the incidence of preeclampsia in our study was also lower and probably because our definition of preeclampsia did not include gravidic hypertension. As Keil et al., we only included term pregnancies in order to better evaluate the real impact on overall maternal weight gain in pregnancy complications. A more recent study showed that a weight gain below recommendations in super obese women did not increase the incidence of severe or moderate prematurity. However, authors stated that the statistical power of their study did not allow them to answer the question, as the frequency of this event was only 3.6% in the studied population. The few studies focusing on pregnancy outcomes in obese women and including our study, which only focused on obese class II women, show that the incidence of macrosomia was decreased in pregnant women with a weight gain below IOM guidelines. However, none of these studies were able to evaluate accurately the impact of a reduction in macrosomia incidence on maternal and neonatal complications such as shoulder dystocia, per partum neonatal asphyxia and post partum hemorrhage. However, it seems that the decrease in macrosomia incidence is itself beneficial, as we know that macrosomia is associated with higher risk of diabetes mellitus, obesity and metabolic dysfunctions later in life [18].

## Conclusion

In pregnant women with class II obesity, a weight gain below IOM guidelines reduced the risk of macrosomia, while it did not increase the risk of SGA. Further studies are necessary to evaluate the impact of this low weight gain on other neonatal parameters and long-term complications.

## Supporting information

S1 Dataset Data PlosOne. (XLS)
